# Hybrid liposomes showing enhanced accumulation in tumors as theranostic agents in the orthotopic graft model mouse of colorectal cancer

**DOI:** 10.1080/10717544.2018.1475517

**Published:** 2018-05-23

**Authors:** Masaki Okumura, Hideaki Ichihara, Yoko Matsumoto

**Affiliations:** Division of Applied Life Science, Graduate School of Engineering, Sojo University, Nishi-ku, Kumamoto, Japan

**Keywords:** Hybrid liposome, theranostics, colorectal cancer, chemotherapy, apoptosis, cancer detection, *in vivo*

## Abstract

Hybrid liposomes (HLs) can be prepared by simply sonicating a mixture of vesicular and micellar molecules in a buffer solution. This study aimed to elucidate the therapeutic effects and ability of HLs to detect (diagnosis) cancer in an orthotopic graft mouse model of colorectal cancer with HCT116 cells for the use of HLs as theranostic agents. In the absence of a chemotherapeutic drug, HLs exhibited therapeutic effects by inhibiting the growth of HCT116 colorectal cancer cells *in vitro*, possibly through an increase in apoptosis. Intravenously administered HLs also caused a remarkable reduction in the relative cecum weight in an orthotopic graft mouse model of colorectal cancer. A decrease in tumor size in the cecal sections was confirmed by histological analysis using HE staining. TUNEL staining indicated an induction of apoptosis in HCT116 cells in the orthotopic graft mouse model of colorectal cancer. For the detection (diagnosis) of colorectal cancer by HLs, the accumulation of HLs encapsulating a fluorescent probe (ICG) was observed in HCT116 cells in the *in vivo* colorectal cancer model following intravenous administration. These data indicate that HLs can accumulate in tumor cells in the cecum of the orthotopic graft mouse model of colorectal cancer for a prolonged period of time, and inhibit the growth of HCT116 cells.

## Introduction

The mortality rate associated with cancer is high worldwide. Colorectal cancer, in particular, is associated with a high mortality rate, with the number of reported deaths being over 700,000 in 2012 (Ferlay et al., 2015). Surgical resection is most commonly used for the treatment for the earlier-stage colorectal cancer. However, surgical resection of the cancer also often results in the removal of healthy tissue during surgery.

5-Fluorouracil (5-FU) has been used as a chemotherapeutic agent for the treatment of colorectal cancer (Longley et al., 2003). FOLFOX (folinic acid, 5-FU, and oxaliplatin) is a 5-FU-based combination of chemotherapeutic drugs used to treat colorectal cancer and is known to improve the survival rate of colorectal cancer patients (André et al., 2004; Bokemeyer et al., 2011; Taieb et al., 2017). However, the side-effects of combination chemotherapy are much more severe than those observed when each chemotherapeutic agent is used singly (André et al., 2004; Taieb et al., 2017). Therefore, the development of chemotherapeutic agents with minimal side-effects is desired to improve the quality of life of the patients.

Advanced colorectal cancer often causes metastases to the lung, liver, and lymph nodes. Metastatic lesions in advanced colorectal cancer can be detected using medical imaging tests, such as positron emission tomography (PET) and magnetic resonance imaging (MRI). However, PET and MRI are both associated with the issues of radiation exposure and high cost.

Theranostics is a treatment strategy that combines therapeutics with diagnostics. The ideal theranostic agents for cancer should have several of the following advantages: (1) theranostic medicines should have the ability to selectively accumulate in the diseased tissue in cancer, (2) a theranostic medicine should be able to mediate a selective therapeutic effect, and (3) theranostic medicines should be safe and nontoxic. Indocyanine green (ICG) is a fluorescent probe that has been widely used in biomedical applications (Maarek et al., 2004; Hoekstra et al., 2013; Vos et al., 2014; De Gasperi et al., 2016). ICG has the attractive properties of very low toxicity and a high absorbance in the range of 600–900 nm (Baohong et al., 2004), and the tissues exhibit transparency on illumination with light in this wavelength range (Maarek et al., 2004; Hoekstra et al., 2013; Vos et al., 2014; De Gasperi et al., 2016). A carrier containing ICG as a fluorescent probe with a high selectivity for cancer could be effectively used to detect cancer.

The effective use of liposomes as carriers in a drug delivery system (DDS) has been widely reported. Hybrid liposomes (HLs) are composed of vesicular and micellar molecules that can be prepared simply by sonication of those molecules in a buffer solution without contamination by organic solvents (Ueoka et al., 1985, 1988). In a DDS study, the therapeutic effects of the anticancer drug, 1,3-bis(2-chloroethyl)-1-nitrosourea, encapsulated into HLs composed of L-ɑ-dimyristoylphosphatidylcholine (DMPC) and polyoxyethylene (20) sorbitan monolaurate (Tween 20) has been shown in an *in vivo* rat model of meningeal gliomatosis (Kitamura et al., 1996). In contrast, HLs composed of DMPC and polyoxyethylenedodecyl ether, without encapsulated anticancer drugs, was shown to inhibit the growth of various type of tumor cells and inducing apoptosis both *in vitro* (Matsumoto et al., 1999, 2005; Iwamoto et al., 2005; Nagami et al., 2006; Komizu et al., 2011) and *in vivo* (Shimoda et al., 2009; Ichihara et al., 2012, 2015). The therapeutic effects of HLs not containing encapsulated drugs, as an anticancer drug in patients with lymphoma has been reported (Ichihara et al., 2008).

This study aimed to elucidate the therapeutic effects of HLs and their ability to detect (diagnose) cancer cells in an *in vivo* orthotopic graft mouse model of colorectal cancer for considering the use of HLs as theranostic agents.

## Materials and methods

### Preparation of HLs

HLs were prepared by sonication of a mixture containing 90 mol% DMPC (NOF Co, Ltd., Tokyo, Japan) and 10 mol% polyoxyethylene (25) dodecyl ether (C_12_(EO)_25_, Nikko Chemicals Co., Ltd., Tokyo, Japan,) in 5% glucose solution using a bath type sonicator (Ultrasonic-Cleaner- WT-200-M, Tokyo, Japan) at 45 °C and a power setting of 200W; the HLs were filtered using a 20 μm cellulose acetate filter (Advantec, Tokyo, Japan).

### Dynamic light scattering measurements

The diameter of the HLs was measured on a light scattering spectrometer (ELSZ-0, Otsuka Electronics, Osaka, Japan) using a He-Ne laser (633 nm) at a 90° scattering angle. The hydrodynamic diameter (*d*_hy_) was calculated using the Stokes-Einstein formula (Equation (1)), where *κ* is the Boltzmann constant, *T* is the absolute temperature, η is the viscosity, and *D* is the diffusion coefficient:

(1) $$d_{\text{hy}}=\kappa T/\text{3}\pi \eta D.$$

### Cell culture

The human colon carcinoma (HCT116) cell line was purchased from the American Type Culture Collection (Manassas, VA). HCT116 cells were maintained in RPMI 1640 medium (Gibco Gaithersburg, MD) supplemented with 100 U/mL penicillin, 50 μg/mL streptomycin, and 10% fetal bovine serum (FBS, HyClone Laboratories, South Logan, UT). The cells were cultured in a 5% CO_2_ humidified incubator at 37 °C.

Normal mouse colon cells were obtained from healthy mice following a previously reported method (Quaroni et al., 1979; Hoffman & Kuksis, 1980) and cultured in RPMI 1640 medium supplemented with 100 U/mL penicillin, 50 μg/mL streptomycin, and 10% FBS.

### Assessment of the 50% inhibitory concentration (IC_50_) of HLs *in vitro*

The 50% inhibitory concentration (IC_50_) of HLs with respect to the growth of HCT116 and normal colon cells was determined using a WST-8 [2-(2-methoxy-4-nitrophenyl)-3-(4-nitrophenyl)-5-(2,4-disulfophenyl)-2H tetrazolium, monosodium salt] assay (Cell Counting Kit-8, Dojindo Laboratories, Kumamoto, Japan). HCT116 and normal colon cells (5.0 × 10^4^ cells/mL) were seeded into 96-well plates and cultured in a 5% CO_2_ humidified incubator at 37 °C for 24 h. Cells were then cultured for a further 48 h after adding DMPC (0.025–2.0 mM), and HLs (0.025–2.0 mM, on the basis of DMPC concentration). The WST-8 solution was added and the cells were incubated for a further 3 h. The absorbance at 450 nm was measured on a spectrophotometer (Molecular Devices, San Jose, CA). The inhibitory effects of HLs on the growth of tumor cells were evaluated by determining *A*_mean_/*A*_control_, where *A*_mean_ and *A*_control_ denote the absorbance of water-soluble formazan in the presence and absence of HL, respectively.

### Analysis of apoptosis by flow cytometry

HCT116 cells were seeded at a density of 5.0 × 10^4^ cells/mL in 60-mm dishes and incubated in a humidified atmosphere of 5% CO_2_ at 37 °C for 24 h. DMPC (0.1–0.6 mM) and HLs (0.1–0.6 mM, on the basis of the DMPC concentration) were added to each dish and the dishes were further incubated for 48 h. The cells were harvested by centrifugation and then resuspended in phosphate-buffered saline (PBS(−)) containing 1 mg/mL RNase, 0.1% Triton X-100, and 40 μg/ml propidium iodide (PI, Molecular Probes, Eugene, OR) in a dark room. The percentage of apoptotic cells was analyzed using a flow cytometer (Epics XL system II, Beckman Coulter, CA).

### Fluorescence depolarization method

The membrane fluidity of intact HCT116 cells was evaluated by the fluorescence depolarization method using a fluorescent probe, 1,6-diphenyl-1,3,5-hexatriene (DPH) (Nacalai Tesque, Japan) (Liebes et al., 1981; Komizu et al., 2006). After pre-incubation of the cells without HLs for 24 h, the cells were treated with 0.05% trypsin/EDTA and resuspended in PBS (−), and then DPH (0.1 μM) was added to the cell suspension (1.0 × 10^6^ cells/mL). The change in cell membrane fluidity for the DPH-labeled HCT116 cells (2.5 × 10^5^ cells/mL) after treatment with HLs was evaluated. After labeling HCT116 cells with DPH, the fluorescence polarization (*P*) value was measured using a fluorescence spectrophotometer (F-7100, HITACHI, Co. Ltd., Tokyo, Japan).

### Assessment of the therapeutic effects of HLs *in vivo*

All mice were handled in accordance with the guidelines for animal experimentation set out by Japanese law. The animal studies were approved by the Committee on Animal Research of Sojo University. BALB/c-R/J mice were kindly provided by Prof. Okada (Kumamoto University, Japan). The mice were bred using a 100% freshly ventilatory change of 14 times per every hour at a room temperature of 25 ± 1 °C, a humidity of 50 ± 10%, and a light/dark cycle of 12 h. The mice were randomly grouped on the basis of the body weight by the stratified randomization method. There were five mice in each group. HCT116 cells (5.0 × 10^6^ cells in 50 μL of Matrigel) were orthotopically implanted into the cecum of anesthetized mice. HLs (dose: 136 mg/kg for DMPC) were intravenously administered once each day for 28 days starting one day after the inoculation with HCT116 cells. Cecum samples were harvested from anesthetized mice after completion of the HLs administration regime and weighed.

### Histological analysis

The cecum samples were harvested from the anesthetized orthotopic graft mouse model of colorectal cancer after completion of the HLs administration regime, and fixed in 10% formalin solution. The cecum samples were embedded in paraffin and sectioned at a thickness of 5 μm. The cecal sections were stained with hematoxylin and eosin (HE) and observed under a light microscope (BZ-X700, Keyence, Osaka, Japan).

### TUNEL method *in vivo*

The detection of apoptotic cells was performed by the TUNEL method using an ApopTag peroxidase *in situ* apoptosis detection kit (S7100, Merck Millipore, Darmstadt, Germany), according to the manufacturer’s directions. After treatment with the HLs, the cecum tissues were harvested from the anesthetized orthotopic graft colorectal cancer mouse model and fixed in 10% formalin solution. Paraffin-embedded sections were then prepared, and apoptotic cells were detected as described above. The cecum sections, including tumors, were stained with the 3,3′-DAB chromogen and observed using an optical microscope.

### Fusion and accumulation of HLs into the cell membrane

The fusion and accumulation of HLs containing a fluorescent probe (Indocyanine Green; ICG, Tokyo Chemical Industry Co., Ltd., Tokyo, Japan) into the membrane of HCT116 cells was performed using a fluorescence cell imaging system (EVOS FL; Thermo Fisher Scientific Inc., Waltham, MA). HCT116 and normal colon cells were seeded at a density of 5.0 × 10^4^ cells/mL in a glass bottomed dish and incubated in a 5% CO_2_ humidified incubator at 37 °C for 24 h. The cells were then treated with HLs containing a fluorescent probe (DMPC = 100 µM; [C_12_(EO)_25_] = 11.3 µM; and [ICG] = 1.1 µM) for 150 min, and the nuclei of cells were also stained with Hoechst 33342 (Molecular Probes, Inc., Eugene, OR) solution for 30 min. The stained cells were observed using a fluorescent cell imaging system (710/40 nm Excitation; 775/46 nm Emission).

### Accumulation of HLs into the tumors of an orthotopic graft mouse model

The accumulation of HLs containing ICG (HL/ICG) in tumors in the orthotopic graft mouse model was observed noninvasively using a fluorescent macroscopic *in vivo* imaging system (Excitation: 725–825 nm, Emission: 790–900 nm, AEQUORIA, Hamamatsu Photonics K.K., Hamamatsu, Shizuoka, Japan). HCT116 cells (5.0 × 10^6^ cells in 50 μL of Matrigel) were inoculated into the cecum. HL/ICG (Dose: 136 mg/kg for DMPC) was intravenously once administered at 28 days day after HCT116 cells were inoculated in the cecum. After the injection of HL/ICG, the bio-distribution of HL/ICG was assessed at each time point (0.5, 2, 24, and 48 h) using a fluorescence macroscopic *in vivo* imaging system. The cecum tissues were harvested from the anesthetized orthotopic graft mouse colorectal cancer model 48 h after treatment with the HLs and observed under a fluorescent microscope.

### Statistical analysis

All data are presented as the mean ± S.D. Data were statistically analyzed using Student’s *t*-test. A *p* value of less than 0.05 was considered to represent a statistically significant difference.

## Results and discussion

### Physical properties of HLs

**Figure 1.**
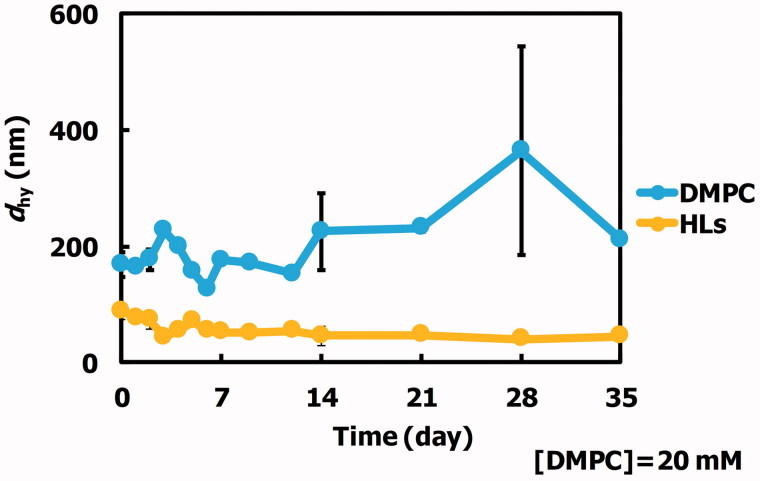
Time courses of *d*_hy_ changes for HLs at 25 °C. The values are represented as the mean ± S.E. Arrow: precipitation.

We examined the morphology of HLs composed of 90 mol% DMPC and 10 mol% C_12_(EO)_25_ using dynamic light scattering measurements, and the results are shown in Figure 1. The hydrodynamic diameter (*d*_hy_) of the HLs was less than 100 nm. HLs were stable for more than one month. So, HLs could be advantageously stored for a long-term period at room temperature (25 °C). In contrast, the *d*_hy_ of DMPC liposomes was over 200–300 nm. DMPC liposomes were unstable. It is noteworthy that HLs with a diameter under 100 nm can avoid the reticular endothelial system (Allen et al., 1991), and therefore should be appropriate for intravenous injection for both *in vivo* and clinical applications.

### The inhibitory effects of HLs on the growth of human colorectal cancer cells

**Figure 2.**
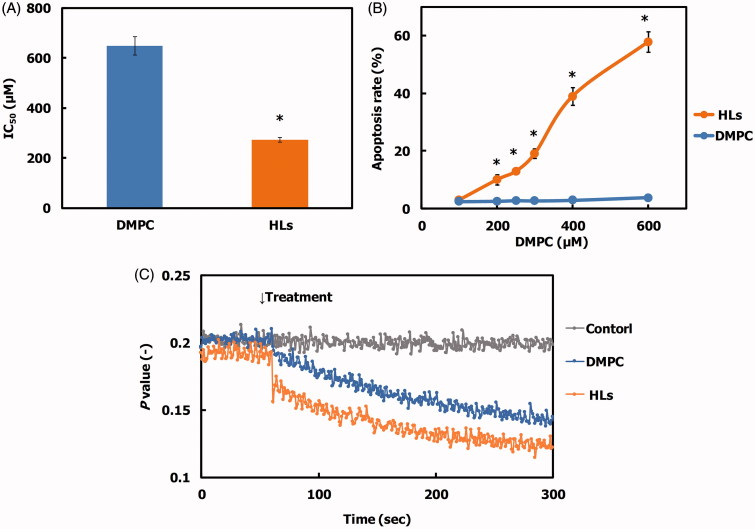
(A) 50% inhibitory concentration (IC_50_) for HLs and DMPC liposomes on the growth of HCT116 cells after 48 h. The values represent the mean ± S.E. *p < .05 (vs. DMPC). (B) Apoptosis rate for HCT116 cells treated with HLs for 48 h. The values represent the mean ± S.E. *p < .05 (vs. DMPC). (C) Time course of fluorescence polarization (*p* value) changes for DPH-labeled HCT116 cells after treatment with HLs.

We evaluated the inhibitory effects of HLs on the growth of human colorectal cancer (HCT116) cells using a WST-8 assay, and the results are shown in Figure 2(A). The 50% inhibitory concentration (IC_50_) value for DMPC liposomes for the growth of HCT116 cells was 648 µM. In contrast, HLs remarkably inhibited the growth of HCT116 cells with an IC_50_ value of 272 µM, which was one-third of that of the DMPC liposomes. These data indicate that greater inhibitory effects were observed on using for HLs compared with those observed on using the parental DMPC liposomes. In contrast, the IC_50_ for HLs with respect to the growth of normal colon cells was 522 µM, which was almost two times higher than that of HCT116 cells (272 µM). Morphological change in human promyelocytic leukemia (HL-60) cells after treatment with HLs composed of phospholipids having the same hydrophilic head group (phosphatidylcholine group) and different hydrophobic alkyl chains (L-α-dilauroylphosphatidylcholine (C12: DLPC), DMPC (C14), L-α-dipalmitoylphosphatidylcholine (C16: DPPC)) and C_12_(EO)_23_ have been investigated using a time-laps video (Nagami et al., 2006). The formation of bleb and corpuscle indicating characteristic feature of apoptosis was observed for HLs of 90 mol% DMPC/10 mol% C_12_(EO)_23_. On the other hand, swelling of cells and dissolving of cell membrane, that is necrosis, were observed for HLs of 90 mol% DLPC/10 mol% C_12_(EO)_23_. Neither apoptosis nor necrosis was observed for HLs of 90 mol% DPPC/10 mol% C_12_(EO)_23_. Thus, two methylene groups of acyl chains in phosphatidylcholines could distinguish between apoptosis and necrosis has already been reported (Nagami et al., 2006). It is noteworthy that greater inhibitory effects of HLs toward human colonic cancer (HCT116) cells were observed without affecting the growth of normal colon cells.

### DNA fragmentation by HLs in human colorectal cancer cells

We analyzed the apoptosis rate in HCT116 cells treated with HLs by flow cytometry, and the results are shown in Figure 2(B). A dose-dependent increase in the apoptosis rate was observed for HCT116 cells treated with HLs, and the apoptosis rate increased to 60%. In contrast, the apoptosis rate for HCT116 cells treated with DMPC liposomes was 2%. These results indicate that HLs induce apoptosis and inhibit the growth of HCT116 cells.

### Changes in the membrane fluidity of HCT116 cells by HLs

We examined the membrane fluidity of HCT116 cells after treatment with HLs using the fluorescent lipid probe, DPH, and the data are shown in Figure 2(C). The fluorescence depolarization (*p* value) of the HCT116 cells treated with HLs was significantly decreased compared to that of control cells and cells treated with DMPC liposomes. The membrane fluidity of the DMPC liposomes was less than that of the HLs (Komizu et al., 2006). These data suggest that the increase in the membrane fluidity could be related to early events in HL-induced apoptosis.

### Therapeutic effects of HLs in an orthotopic graft mouse model of colorectal cancer

**Figure 3.**
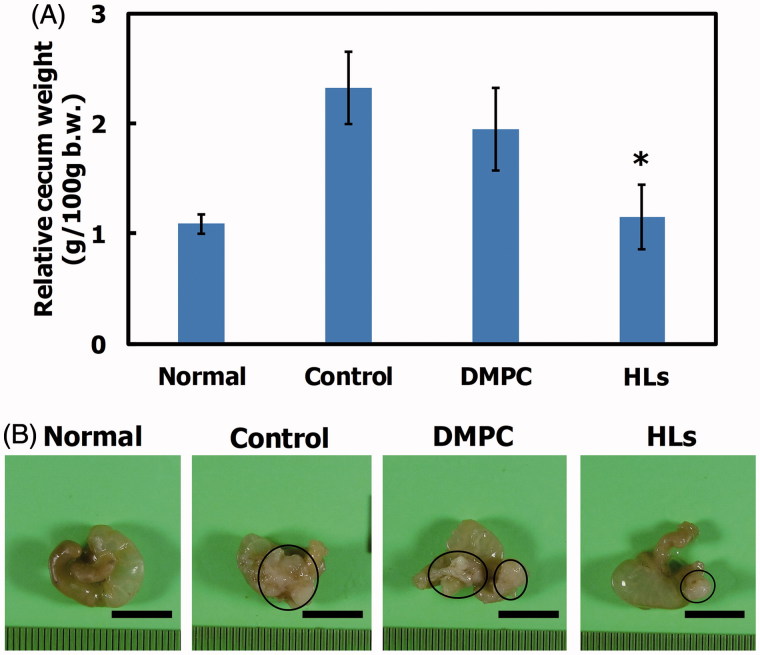
(A) Relative cecum weight of the orthotopic graft model mice treated with HLs after inoculation with HCT116 cells in the cecum. The values represent the mean ± S.E. *p < .05 (vs. control, DMPC). (B) Photographs of cecum in the orthotopic graft model mice treated with HLs after inoculation of HCT116 cells to the cecum. Circle: tumor. Scale bar: 1 cm.

We have reported the therapeutic effects of Hs using hepatic metastasis xenograft mouse models of colorectal cancer (WiDr and HCT116) cells *in vivo* (Ichihara et al., 2012, 2014). So, we investigated the therapeutic effects of HLs in an orthotopic graft mouse model of colorectal cancer with HCT116 cells, and the results are shown in Figure 3. HCT116 cells were orthotopically transplanted into the cecum of the BALB/c-R/J mice. Following this, HLs were intravenously administered into the orthotopic graft mouse model of colorectal cancer once a day for 28 days from the first day following HCT116 cell transplantation. The mouse models of the advanced cancer by surgical orthotopic implantation of a tumor tissue in a treatment experiment for colorectal cancer have also been used (Furukawa et al., 1993; Hoffman, 1999, 2017; Itoh et al., 1999). In this study, we used mouse models of orthotopic injection of HCT116 cell suspensions to examine theranostic effect of HLs toward early stage primary colorectal cancer. As shown in Figure 3(A), the relative cecum weight of the mouse model treated with HLs significantly decreased compared to that of control mice and mice treated with DMPC liposomes, and was similar to that of normal mice. A remarkable reduction in the weight of cecum tumors was observed in the model mice treated with HLs, while an enlargement in the tumor size was observed in the cecum of control mice and mice treated with DMPC liposomes (Figure 3(B)). These data highlight the remarkable therapeutic effect of HLs, even in the absence of an anticancer drug, in this orthotopic graft mouse model of colorectal cancer.

### Reduction in tumor area in the cecum of orthotopic graft mouse model of colorectal cancer after treatment with HLs

**Figure 4.**
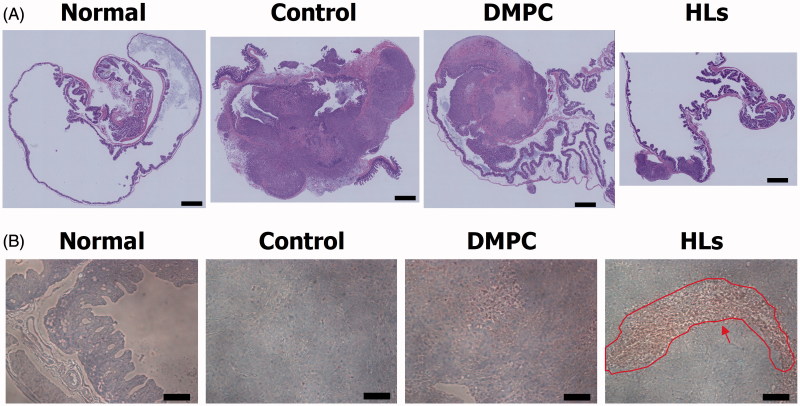
(A) Micrographs of HE staining of the cecum in the orthotopic graft model mice after treatment with HLs. Scale bar: 1 mm. (B) Micrographs of TUNEL staining of cecum tissue sections from the orthotopic graft model mice after treatment with HLs. Arrows: apoptotic cells. Scale bar: 100 μm.

We also assessed the therapeutic effects of HLs in the orthotopic graft mouse model of colorectal cancer with HCT116 cells on the basis of the histological analysis. HLs were intravenously administered once each day for 28 days from the day after inoculating HCT116 cells into mice. The cecum was harvested from the anesthetized mice immediately after the last treatment with HLs; the cecum samples were analyzed by hematoxylin and eosin (HE) staining. The results are shown in Figure 4(A). Large tumors were observed in the cecum of control mice and mice treated with DMPC liposomes. In contrast, very small tumors were observed in the cecum samples of mice treated with HLs, which were fairly similar to the normal cecum samples. HLs have not also been shown side-effects using healthy rats and mice *in vivo* (Ichihara et al., 2008; Shimoda et al., 2009). HLs were metabolized in the livers after intravenous administration to healthy mice as already described (Ichihara et al., 2012). These data indicate that intravenous treatment with HLs could be effective in inhibiting the enlargement of tumors in the orthotopic graft mouse model of colorectal cancer.

### Induction of apoptosis by HLs for orthotopic graft model mice of colorectal cancer

We next examined the mechanism of the therapeutic effect of HLs in the cecal tumors from the mouse orthotopic graft model by performing TUNEL analysis. HLs were intravenously administered to the orthotopic graft mouse model of colorectal cancer once a day for 28 days beginning the day after the inoculation with HCT116 cells. We evaluated the induction of apoptosis by HLs using TUNEL staining, and the results are shown in Figure 4(B). Numerous apoptotic cells were observed in the tissue sections of cecal tumors after the intravenous administration of HLs. In contrast, apoptotic cells were not observed in the cecums from normal mice, control mice, and mice treated with DMPC. These results indicate that intravenous injection of HLs inhibits the growth of HCT116 cells due to the induction of apoptosis. The pathways of apoptosis induced by HLs in human promyelocytic leukemia (HL-60) *in vitro* have already been reported (Matsumoto et al., 2005). HL fused and accumulated in HL-60 cell membranes. The apoptotic signal first passed through the mitochondria, then caspase-9 and caspase-3, and then reached the nucleus. Second, the apoptotic signal passed through FAS, then caspase-8 and caspase-3, and then reached the nucleus. Therefore, the apoptotic signal by HLs for HCT116 cells could pass through mitochondria and activation of caspase-9, -8, and -3, and then reach the nucleus following these two pathways *in vivo*.

### Selective accumulation of HLs in HCT116 cells *in vitro*

**Figure 5.**
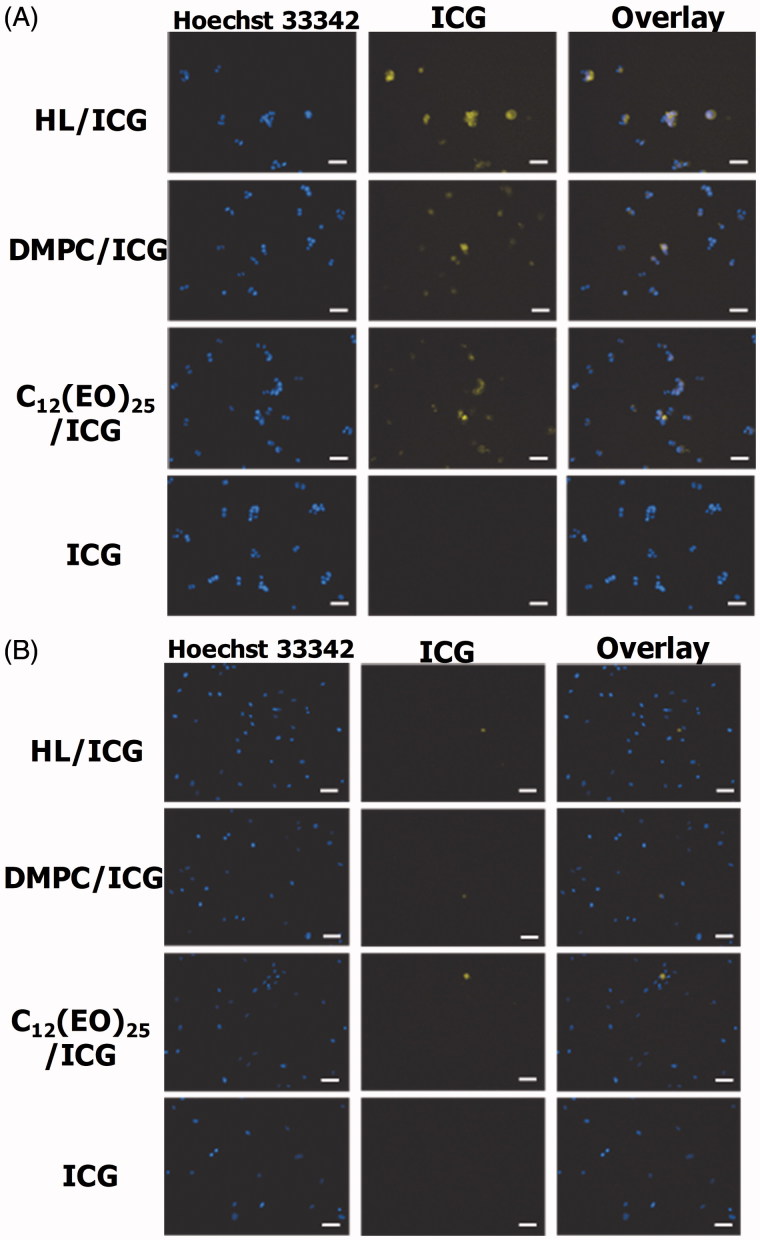
(A) Fluorescence micrographs of HCT116 cells after treatment with HL/ICG for 3 h. (B) Fluorescence micrographs of normal colon cells after treatment with HL/ICG for 3 h. Scale bar: 50 μm.

We next examined the accumulation of HLs encapsulating ICG (HL/ICG) in HCT116 cells by fluorescence microscopy, and the data are shown in Figure 5(A). There was a remarkable accumulation of HL/ICG in the HCT116 cells, although lower levels of accumulation of DMPC/ICG and C_12_(EO)_25_/ICG were observed. ICG alone did not accumulate in HCT116 cells.

Next, we assessed the accumulation of HL/ICG in normal colon cells, and the data are shown in Figure 5(B). HL/ICG did not accumulate in normal colon cells. The membrane fluidity of tumor cells is considered to be higher than that of normal cells (Papahadjopoulos et al., 1973; Deliconstantinos, 1987; Sok et al., 1999; Komizu et al., 2011; Komizu et al., 2012). These results suggest that there is selective accumulation (useful for diagnosis) of HL/ICG only in colorectal cancer cells that have a greater degree of membrane fluidity compared to normal colon cells.

### Accumulation of HLs in tumors for prolonged periods of time *in vivo*

**Figure 6.**
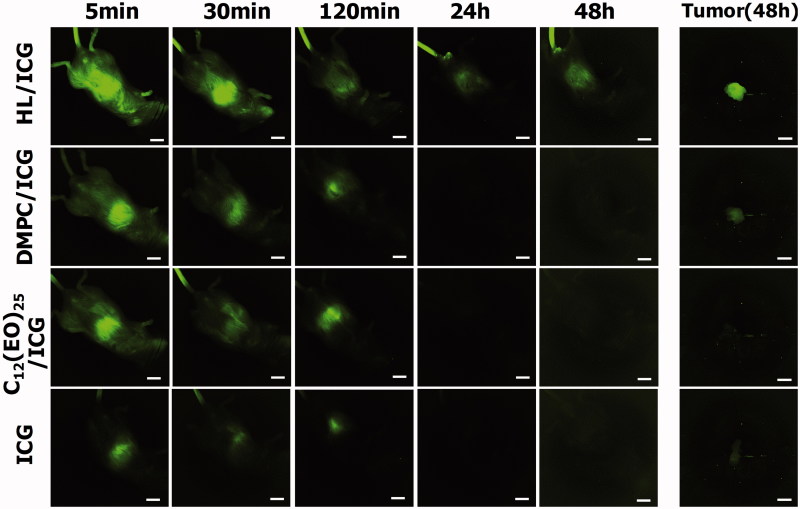
Long-term accumulation of HLs encapsulating ICG in the tumors in the orthotopic graft model mice four weeks after inoculation with HCT116 cells. Scale bar: 1 cm.

We next assessed the accumulation of HL/ICG in tumors in the orthotopic graft mouse model of colorectal cancer using an *in vivo* fluorescence imaging system. HCT116 cells were orthotopically transplanted into the cecum of the BALB/c-R/J mice. Following this transplantation, HL/ICG was intravenously just once administered at 28 days day after HCT116 cells were inoculated into the cecum of mice. The accumulation of HL/ICG in tumors was investigated using a macroscopic fluorescent *in vivo* imaging system, and the data are shown in Figure 6. The ICG green fluorescence had disappeared within 24 h after the injection of DMPC/ICG, C_12_(EO)_25_/ICG, or ICG alone. ICG has been used to evaluate liver function in clinical practice because ICG is immediately removed from circulation through the liver (Hoekstra et al., 2013; Vos et al., 2014; De Gasperi et al., 2016). Based on this it would be expected that DMPC/ICG, C_12_(EO)_25_/ICG, and ICG alone would be excreted from the liver into the intestine. In contrast, green ICG fluorescence was observed in the cecum area of the orthotopic graft mouse model of colorectal cancer 48 h after the intravenous injection of HL/ICG. Furthermore, a strong green fluorescence, indicating the accumulation of HL/ICG, was observed in the tumors resected from mice at 48 h after administration of anesthesia. These data indicate that HLs can accumulate in the tumor cells in the cecum in this mouse model of colorectal cancer for a prolonged period of time. It is noteworthy that a highly sensitive detection of tumors using HL/ICG was achieved in this orthotopic graft mouse model of colorectal cancer; this may enable diagnosis of tumors.

## Conclusion

We have established, for the first time, that HLs have a therapeutic effect and can detect tumors in an orthotopic graft mouse model of colorectal cancer. The HLs appear to exert their therapeutic effect by directly inhibiting the growth of HCT116 cells, in the absence of any chemotherapeutic agent, perhaps by stimulating apoptosis *in vitro*. Remarkable reduction in the relative cecum weight was seen in the orthotopic graft mouse model of colorectal cancer intravenously administered with HLs. The decrease in the tumor size in the cecal sections was also observed during histological analysis involving HE staining. Intravenous HLs administration also caused an induction of apoptosis in the HCT116 cells in the cecum of the orthotopic graft model mice, as assessed by TUNEL staining.

With regard to the detection (diagnosis) of colorectal cancer by HLs, a selective accumulation of HL/ICG (which contained the encapsulated ICG fluorescent probe) was observed in the colorectal cancer cells, which have a high degree of membrane fluidity, while accumulation was not observed in normal colon cells. Accumulation of HL/ICG in the tumor cells in the cecum samples from the orthotopic graft mouse model of colorectal cancer was observed after the intravenous administration of HL/ICG *in vivo*. The data in this study suggest that, in the future, HLs could potentially be used as theranostic agents in patients with colorectal cancer, possibly through photodynamic therapy.
